# Surgical Management of Inframammary Hidradenitis Suppurativa with Reduction Mammaplasty Technique: A Report of Two Cases

**DOI:** 10.3390/reports9020177

**Published:** 2026-06-06

**Authors:** Enrico Caporali, Paolo Toninello, Monia Maritan, Alessandro Gatti, Giorgio Berna

**Affiliations:** 1 Department of Plastic Surgery, Hospital Ca’ Foncello, AULSS2 Marca Trevigiana, 31100 Treviso, Italy; toninello.paolo@gmail.com (P.T.); monia.maritan1@aulss2.veneto.it (M.M.); giorgio.berna@aulss2.veneto.it (G.B.); 2 Department of Dermatology, Hospital Ca’ Foncello, AULSS2 Marca Trevigiana, 31100 Treviso, Italy; alessandro.gatti1@aulss2.veneto.it

**Keywords:** hidradenitis suppurativa, inframammary fold, breast reduction, surgical management, multidisciplinary care

## Abstract

**Background and Clinical Significance**: Hidradenitis suppurativa (HS) is a chronic, debilitating skin disorder that often affects the inframammary fold (IMF). While surgical management, particularly wide local excision (WLE), is the gold standard for severe cases, less is known about the role of breast surgery techniques in treating HS in this area; **Case Presentation**: This report presents two cases of female patients with bilateral inframammary HS and mammary hypertrophy, both treated with reduction mammaplasty to excise diseased tissue while addressing breast volume and contour. Both patients had experienced inadequate response to medical therapies, including biologic treatments, and presented with distinct clinical features—one with significant asymmetry and active disease, and the other with more scarring and hypertrophic lesions. During follow-up, no recurrence of disease was observed and both patients reported improved breast appearance and satisfaction; **Conclusions:** These cases underscore the importance of a multidisciplinary approach, with dermatologists and surgeons both playing a key role in the management of this complex condition.

## 1. Introduction and Clinical Significance

Hidradenitis suppurativa (HS) is a chronic and disabling inflammatory skin disease arising from the pilosebaceous unit. It usually occurs in areas rich in apocrine sweat glands, including the axilla, groin, perineum and, less frequently, the inframammary folds [1,2,3]. It presents as chronic and relapsing flares of deep and painful nodules with abscess formation, purulent discharge and sinus tracts formation; when healed they frequently leave extensive and bridged scarring [3,4].

It affects young individuals between 20 and 40 years and it is more common in the female gender. Pathogenesis of HS is multifactorial, but there is a consensus on its inflammatory etiology driven by a dysfunction of the follicular unit. Alterations in skin microbiota and hormonal assets, obesity and smoking are regarded as main risk factors, with genetic and familial predisposition also playing a role [5,6,7].

Treatment involves the use of systemic and topical antibiotics, oral retinoids and biologics; surgical interventions such as drainage or deroofing are used for early stages, whereas wide local excision is the gold standard in severe forms where medical therapy failed and/or significant scars formed [5,6].

We herein present two cases of young female patients with disabling inframammary forms of HS, both treated with a reduction mammaplasty surgery to ablate the diseased skin and subcutaneous tissue. We then provide a literature review of surgical management of this region. These cases offer exploratory observations on the role of careful surgical indication with emphasis on preoperative planning. It also emphasize the value of multidisciplinary evaluation to ensure multimodal treatment to synergically be as effective as possible.

## 2. Case Presentation

### 2.1. Case Description and Diagnostic Assessment

The first case is a 23-year-old female with a longstanding history of hidradenitis suppurativa since adolescence that was referred to our Clinic by the Dermatology Department of our hospital for surgical management. The patient had a history of recurrent HS flares affecting the neck, groin, buttocks, and inframammary folds.

**Figure 1:**
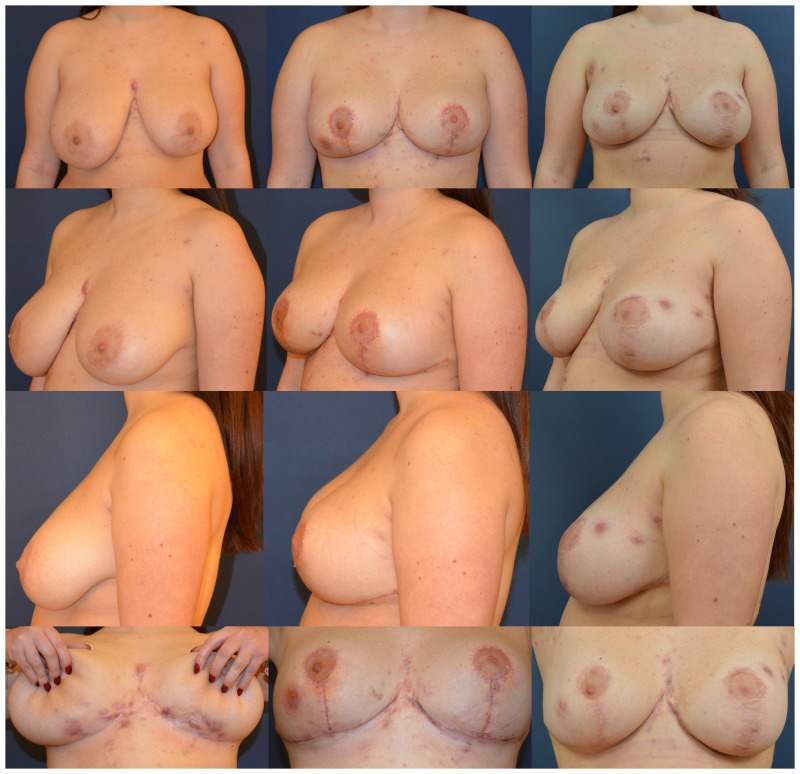
A 23-year-old patient, preoperative (**left**), 3-month (**middle**) and 9-month postoperative (**right**) photographs (the patient provided written consent for the use of these photographs for scientific purposes).

Previous treatments with antibiotics (Doxycycline, Minocycline), oral retinoids (Isotretinoin) and biologic therapy (Adalimumab) turned out to be unsuccessful or were discontinued because of side effects. In the last two years, she had been on Secukinumab 300 mg every 4 weeks with remission of disease except for intermittent flares confined to the inframammary folds. Clinical examination showed bilateral mammary hypertrophy with volume asymmetry and moderate ptosis. The left breast had a sternal notch-to-nipple distance of 25 cm, while the right breast measured 26 cm. Inframammary folds were bilaterally affected by multiple red nodules, abscesses, sinus tracts and extensive scars; lesions were assessed as Hurley III. Also, Dermatology Life Quality Index (DLQI) was administered, displaying a score of 13. Both IMFs showed significant disease involvement, making wide excision without reconstructive intervention a less favorable option due to potential disfigurement (Figure 1).

Preoperative imaging with ultrasound revealed hypoechoic fluid collections with thickened walls of 24 and 36 mm in the inferior quadrants of both breasts, while mammography was negative.

**Figure 2:**
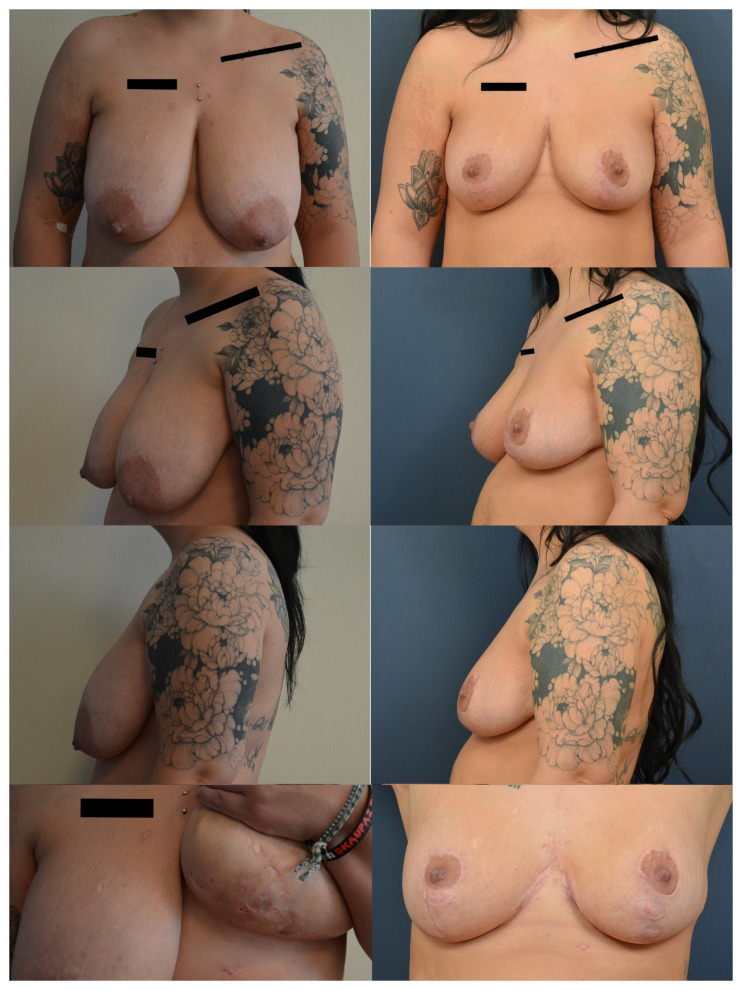
A 32-year-old patient, preoperative (**left**) and 6-month postoperative (**right**) photographs (the patient provided written consent for the use of these photographs for scientific purposes).

The second case is a 32-year-old female with a history of hidradenitis suppurativa that was referred for surgical treatment due to bilateral inframammary disease. She had been managing the disease with Secukinumab 300 mg every two weeks after failing treatment with Adalimumab. The patient had a history of perianal and inguinal HS, both of which had required surgical intervention. Upon clinical examination, the patient presented with bilateral mammary hypertrophy with severe ptosis but without significant asymmetry. The inframammary folds were affected by fibrotic tissue with some hypertrophic scarring, but no acute abscesses or sinus tracts were present. The patient reported discomfort and relapsing pain due to the chronic scarring. The lesions were assessed as Hurley II/III (Figure 2).

As with the first case, preoperative ultrasound showed changes in the inferior breast quadrants, including thickened skin and fibrotic tissue.

### 2.2. Therapeutic Intervention

Surgery was planned only after achieving adequate disease control and clinical stabilization. In our clinical practice, remission prior to surgery is defined according to HiSCR50 criteria as at least 50% reduction in abscess and inflammatory nodules count, without an increase in abscesses or draining fistulas. Clinical judgment and multidisciplinary evaluation were also considered in determining surgical candidacy.

Oral antibiotics were started before surgery to reduce the bacterial burden of the abscesses. Surgical planning involved an inverted-T reduction mammaplasty to excise the diseased tissue, address the ptosis and correct asymmetry if present. While the patient was standing up straight, preoperative markings were drawn: midline and breast meridians were marked and the future nipple position was pointed around 1 cm above the anterior projection of the inframammary fold; the areolar upper extent was placed 2 cm above this first point. From there the pillar lines were drawn: the top areolar marking was connected to the breast meridian at the IMF while pushing the breast medially and laterally, marking the lateral and medial pillar lines respectively. The dome was drawn to embrace the areolar circumference and the vertical pillars were measured to result 5–6.5 cm.

The horizontal lines were drawn in a lazy S shape with the extremities kept within the breast footprint, while the inferior line was marked at the IMF, between 1 cm above and below its natural position. This variation from the usual markings for reduction mammaplasty allowed us to include the pathologic tissue to be excised within incision markings with at least 1 cm of clinically clean margins.

The incision was made along the inferior markings and carried down to the pectoral fascia. Dissection then proceeded superiorly while preserving a superior dermoglandular pedicle, which had been previously de-epithelialized, to maintain vascular supply to the nipple–areola complex (NAC) through the superior breast vascular network (branches from the thoracoacromial artery and second and third intercostal spaces perforators from the internal mammary artery). The pedicle was designed according to the individual breast dimensions and the amount of planned tissue excision, ensuring adequate tissue support and viability of the NAC; it was sculpted full thickness while leaving only a thin layer of fat above the pectoralis fascia. Excess adipoglandular tissue together with the involved skin and subcutaneous tissue was subsequently excised en bloc, including clinically unaffected margins surrounding HS lesions. The volume of tissue removed was adjusted when necessary to obtain symmetry between the breasts. Closure was performed through approximation of the glandular pillars in order to reshape the breast mound and reduce tension on the skin closure and suction drains were positioned bilaterally.

Patients were discharged on oral Doxycycline for 5 days with close outpatient follow-up. No early postoperative wound complications or delayed healing were observed. Suction drains were removed on postoperative day 2 in patient #1 and postoperative day 4 in patient #2.

### 2.3. Outcomes, Follow-Up and Patients’ Perspective

Despite still suffering from occasional breast and trunk flares, no recurrence of inframammary disease was detected at 9 months postoperatively in patient #1. Overall cosmesis was improved with the correction of ptosis and asymmetry although some extent of lower pole lengthening was noted. The patient reported a significant improvement in her daily comfort following surgery (postoperative DLQI was 2) and she was strongly satisfied with the new breast contour. Additionally, she experienced less irritation when wearing a bra, as friction no longer aggravated previously inflamed areas (Figure 1).

At 6-month follow-up, patient #2 had no recurrence of HS in the IMF, and the scarring was improved. The main positive outcome reported by the patient was a substantial reduction in pain; DLQI score was reduced to 3. Overall, she expressed satisfaction with both the cosmetic and functional results (Figure 2).

## 3. Discussion

In moderate and severe forms, HS deeply impacts on the quality of life and psychosocial well-being of patients with important pain disability and higher rates of depression, anxiety and lower mental health being reported [5,6,8]. In this context, the role of surgical management becomes crucial—as advised by the European S2k guidelines—especially when a shift from inflammatory lesions to irreversible tissue destruction occurs, in which the presence of biofilm embedded in scar tissues makes them particularly resistant to medical therapies [9,10]. It is important to plan surgery during the remission of the inflammatory process to improve surgical and postsurgical outcomes [11].

The aims of surgical therapy are to offer symptom relief and ablate irreversibly damaged tissues [6]; however, the choice of the most appropriate surgical approach depends on disease stage, localization and extension of lesions, patients’ comorbidities and previous treatments [11,12]. Lesions at initial stages can benefit from incision and drainage, a procedure that can reduce pain but is burdened with high recurrence rates—especially when the lesions are chronicized [10,11]—and deroofing, a tissue-sparing procedure that removes the roof of abscesses and sinus tracts and debrides them, allowing for secondary healing [12]. In contrast, for Hurley II/III stages wide local excision (WLE) is a broadly accepted method, performed with 1–2 cm of healthy margins and brought down to healthy subcutaneous tissue [2,6,12,13].

Following excision, different reconstructive strategies can be adopted, including primary closure, skin grafting, the use of pedicled flaps or, in some cases, healing by secondary intention [9,12,13]. In 2015 Mehdizadeh et al. [14] published a meta-analysis that compared recurrence rates in different surgical procedures, finding that WLE was the procedure associated with fewer recurrences (13%) when compared with local excision (22%) and deroofing (27%). When WLE was performed, the analysis of the chosen method of closure revealed lower recurrence rates in the skin graft and flap groups, while primary closure was associated with higher percentages of flares.

In hidradenitis suppurativa, the breast is involved in 37% of cases, especially in the female group [15,16]. Inframammary HS is a less common presentation [1,2], historically associated with higher recurrence rates after surgical therapies in comparison with axillary or groin regions [17]; in 2020 a case series by Shavit et al. of 22 patients treated with WLE and secondary intention healing showed an 18% recurrence rate [2]. Higher recurrence rates reflect the anatomy of the breasts, especially when some degree of ptosis is present and the breasts lie against the chest wall: in this situation at the level of the inframammary folds skin temperature rises and the moistness and maceration that form stimulate bacterial growth [1,4,18]. Also, friction between skin surfaces and with tight garments like bras appears to stimulate follicular occlusion and hyperplasia that play an important role in triggering the condition [19].

Moreover, the aesthetic value of the breast in women poses important challenges in the surgical management of inframammary HS, demanding for an effective surgery while preserving the cosmetic appearance of such relevant structures, and this must be considered when planning the wide excision and subsequent reconstruction. When the IMF and/or the NAC are extensively involved, simple WLE produces alteration of breast contour, as seen in some reports [20,21], even resulting in a modified radical mastectomy in a dramatic case reported by Moosa et al. [22].

In our cases, both patients presented with moderate-to-severe breast ptosis and mammary hypertrophy associated with extensive bilateral inframammary involvement.

Given the localization and extension of lesions, conventional wide local excision alone would likely have resulted in substantial contour deformity, displacement of the nipple–areola complex, and potentially unfavorable aesthetic outcomes. Therefore, a Wise-pattern reduction mammaplasty was considered preoperatively based on specific anatomical and disease-related characteristics. This technique, which is well-established in reconstructive breast surgery to treat gigantomastia and to perform oncoplastic procedures, allows NAC uplift and excision of lower breast quadrants and the skin overlying them reshaping the breast mound. It was then selected as a surgical solution capable of integrating oncologic-like principles of complete excision while simultaneously improving breast contour. In our case the anatomy of the patients was favorable since the breasts were of considerable dimensions and patient #1 had asymmetry she wished to correct. Based on our experience, this approach may be applicable in selected patients with inframammary HS associated with mammary hypertrophy and/or moderate-to-severe ptosis. In these individuals, correcting the sagging of the breasts on the chest wall reduces the factors associated with HS flares, such as friction and maceration, thus representing a potential factor in reducing the risk of recurrence [9]. Therefore, reduction mammaplasty or mastopexy may offer a dual benefit by enabling excision of diseased tissue while simultaneously correcting anatomical characteristics potentially involved in the pathophysiology of the disease. On the other hand, in patients without significant ptosis, the role of this approach remains less clear and alternative reconstructive strategies may be more appropriate.

An additional observation from our experience concerns postoperative breast shape: in patient #1 a moderate lower pole lengthening was noted while in our records it is quite uncommon when performing the same technique on healthy patients. We attribute this to a general loss of elasticity and integrity that can occur in the chronically inflamed skin due to structural and biochemical alterations: it has been noted that chronic inflammatory diseases such as HS can cause an uncontrolled remodeling of the extracellular matrix with increased activity of metalloproteinases resulting in a dysregulated collagen deposition and crosslinking [23]. This aspect should be considered in future preoperative planning.

Adoption of such a technique is an effective yet scarcely described in the literature way to address extensive inframammary HS. Williams et al. in 2001 published a case series of five patients who underwent reduction mammaplasty or mastopexy with superomedial pedicle; the series showed no IMF recurrences [4]. In 2010 Nakanishi et al. presented a case of inframammary HS treated with excision of inframammary and periareolar skin in a keyhole pattern; in that case, no dermis pedicle was preserved and NAC vascularization was supported by the mammary tissue beneath, completely maintained in the procedure [1].

In our opinion the last two studies did not specifically focus on the integration of surgery within a comprehensive treatment framework—encompassing both medical and surgical therapies—and therefore do not adequately address the critical aspect of selecting the correct indication and the most appropriate timing for surgery. A multidisciplinary approach with dermatologists and surgeons contributed to a positive outcome: as a matter of fact, this is essential in ensuring optimal disease control before surgery, determining candidacy for surgical intervention, and coordinating medical therapy to minimize disease recurrence postoperatively.

Both cases presented had an extensive and multifocal form of hidradenitis and a history of medical therapy failures; eventually, the treatment with Secukinumab was associated with improved disease control and limited disease recurrence in the IMF, thus allowing for successful surgical intervention. In fact, surgery was performed in a remission phase of the disease, which in our clinical practice is identified as a reduction in the number of abscesses and nodules of at least 50% according to HS Clinical Response (HiSCR) [16]. Secukinumab is a monoclonal antibody directed against IL-17A; this interleukin is overexpressed in HS along with increased levels of Th17 cells, thus providing a rationale for the use of Secukinumab as a therapeutic strategy [9]. At the time these cases were treated, Secukinumab (Cosentyx) was available in Italy only in the form of nominal therapeutic use, since the indication for the treatment of HS was yet to be approved. The biologic drug was administered according to approved prescribing information [9,24], where the recommended dose in adult patients with moderate to severe HS is 300 mg by subcutaneous injection at weeks 0, 1, 2, 3 and 4 and every 4 weeks thereafter. If a patient does not adequately respond, it can be considered to increase the dosage to 300 mg every 2 weeks, as was done in patient #2. Administration of Secukinumab every 14 days was found safe and clinically effective in the SUNSHINE and SUNRISE clinical trials [25].

RCT and real world data report the association of biologics and surgery to be superior to biologics or surgery alone in obtaining a clinical response [9,26,27]: biologic therapy reduces indications and extent of surgery and optimizes surgical outcomes reducing the inflammation, while surgery reduces the inflamed tissue load increasing the effectiveness of biologic therapy in the post-surgical period [9,26].

There is an ongoing debate whether to discontinue biologics or not prior to surgery, as traditionally they have been associated with wound infection and delayed wound healing problems [11,28]. In 2021 the SHARPS trial [29] tested the efficacy and safety of Adalimumab (usually the first line biologic agent in the treatment of HS) in conjunction with surgery, finding that the group receiving adalimumab achieved clinical response more frequently than the placebo group and no increased risk of postoperative wound infection, complications or hemorrhage was observed; on the contrary, some report a significant risk of rebound flares after the discontinuation of therapy [28]. Based upon these observations, current European S2k guidelines advise for continuation of Adalimumab during surgery [9], although multiple expert groups favor the extension of this statement towards all appropriate HS-directed biologics [11,30]. A consensus of Italian HS experts [11] advises for the discontinuation of biologic therapy only in selected cases where a history of infective complications or increased risk of infection exist; in these cases the drug should be interrupted for five times its half-life.

In our cases the biologic therapy was not discontinued nor were changes in the interval between doses made, and surgery was performed between two doses. No complications were observed although we are not able to express general recommendations based on these two cases. Given the limited sample size and multimodal treatment approach, the relative contribution of biologic therapy and surgery to long-term outcomes cannot be determined. Only a single biologic agent (Secukinumab) was used in both cases as part of the multidisciplinary treatment strategy. Although similar considerations may potentially apply to other HS-directed biologic therapies, whether comparable outcomes can be achieved with different biologic agents in combination with surgery remains uncertain, limiting the generalizability of these findings.

## 4. Conclusions

This article offers preliminary observations regarding inframammary hidradenitis suppurativa and a potential surgical management approach in synergy with medical therapy. However, the very limited number of cases and the relatively short follow-up period represent important limitations, and therefore no definitive conclusions can be drawn. Postoperative outcomes were primarily assessed through clinical follow-up and DLQI evaluation, while additional standardized measures were not collected.

Ongoing follow-up of these patients is planned with periodic clinical assessments beyond the first postoperative year to further evaluate long-term disease control and detect possible recurrence. Given the chronic and relapsing nature of HS, the recurrence-free outcomes observed should be interpreted as preliminary findings rather than evidence of durable long-term efficacy.

## Data Availability

The original contributions presented in this study are included in the article. Further inquiries can be directed to the corresponding author.

## Abbreviations

The following abbreviations are used in this manuscript:

HS	Hidradenitis Suppurativa
IMF	Inframammary Fold
DLQI	Dermatology Life Quality Index
HiSCR	Hidradenitis Suppurativa Clinical Response
WLE	Wide Local Excision
NAC	Nipple-Areola Complex
IL-17A	Interleukin-17
Th17	T helper 17 Cells
